# Regression analysis and driving force model building of CO_2_ emissions in China

**DOI:** 10.1038/s41598-021-86183-5

**Published:** 2021-03-24

**Authors:** Yi Zhou, Jinyan Zhang, Shanying Hu

**Affiliations:** 1 Department of Chemical Engineering, Center for Industrial Ecology, Tsinghua University, Beijing, China; 2 PBC School of Finance, Tsinghua University, Beijing, China

**Keywords:** Governance, Climate-change impacts, Sustainability

## Abstract

In recent years, global warming has become increasingly devastating, leading to severe consequences, such as extreme weather events and sea-level rise. To reduce carbon dioxide emissions, it is essential to recognize different emission sources and key driving factors. Three main carbon emission sources from the period between 1990 and 2017 were identified in China: the energy industry, fuel combustion in other industries, and industrial process. For each source, a driving force model was developed via multiple linear regression. Based on these models, forecasts of the carbon intensity and total CO_2_ emissions were obtained from 2018 to 2030. The results demonstrate that the CO_2_ emission intensity and total emissions will continue to decrease but more effort will be required to achieve the goal of Paris Agreement.

## Introduction

Since the industrial revolution, the world has undergone a process of continuous warming. According to the fifth climate change assessment report issued by the UN Intergovernmental Panel on Climate Change in 2014^1^, the global average surface temperature has risen by approximately 0.85 °C. Compared to warming levels worldwide, China is facing a more severe situation.

**Figure 1:**
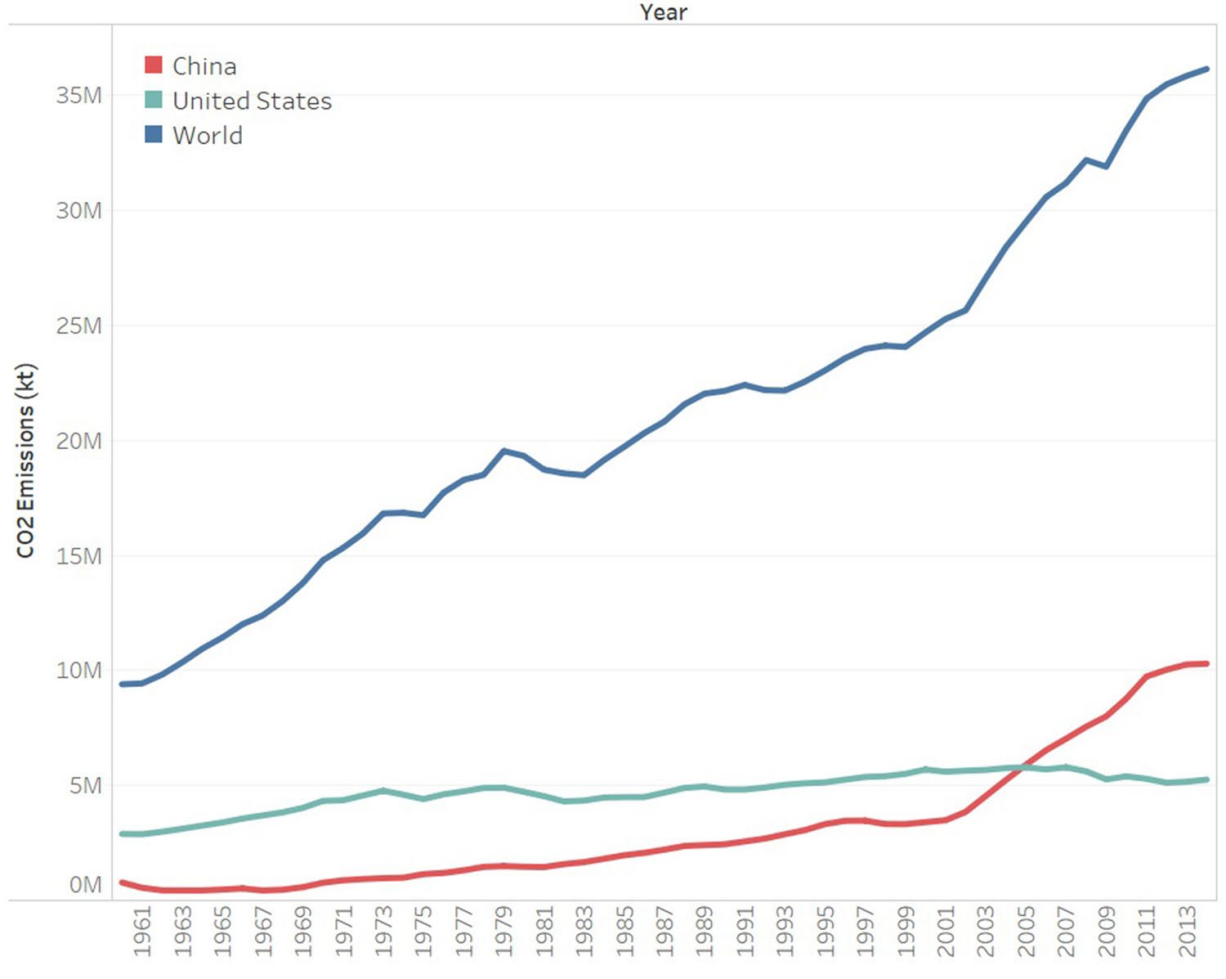
CO_2_ emissions across the globe and, in China, and the United States.

Global warming is mainly caused by greenhouse gas (GHG) emissions. In 2017, the concentrations of the three major GHGs, namely carbon dioxide, methane, and nitrous oxide in the atmosphere were 405.5 ppm, 1859 ppb, and 329.9 ppb, respectively^2^, which were approximately 46%, 157%, and 22% higher, respectively, than the levels before widespread industrialization. To control the ecological deterioration caused by global warming, it is essential to reduce GHG emissions, especially carbon dioxide emissions. Figure 1 shows the trend of carbon emissions across the globe, in China, and in the United States over the past 50 years^3^. Global carbon emissions are experiencing a rapid growth, with 78% of emissions compromising fossil fuel combustion and industrial production process emissions. Since 2005, China has surpassed the United States to become the largest carbon emitter. In 2012, China's total annual carbon emissions exceeded 10 billion tons for the first time.

Faced with the problem of the global warming, various countries and international organizations have started to take action. The United Nations adopted the United Nations Framework Convention on Climate Change in 1992 and the Kyoto Protocol in 1997. In December 2007, the 15th Conference of the Parties (COP 15) to the United Nations Framework Convention on Climate Change (UNFCCC) was held in Copenhagen and negotiators from 192 countries debated global emissions reduction agreements from 2012 to 2020. In December 2015 the Paris Agreement was passed and its long-term temperature goal was to maintain the increase in the global average temperature to well below 2 °C (3.6 °F) above preindustrial levels and to pursue efforts to limit the increase to 1.5 °C (2.7 °F). China has actively participated in global climate change negotiations and has taken responsibility for reducing its emissions. In the 2009 Copenhagen Climate Change Conference, China pledged to reduce carbon dioxide emissions per unit of the GDP (carbon emission intensity) by 40–45% by 2020 over the 2005 and in the U.S.–China Joint Presidential Statement on Climate Change issued in November 2014, it was also proposed that by 2030, the carbon dioxide emissions in China will reach a peak, which should be attained as soon as possible, and the proportion of nonfossil energy in primary energy consumption will increase to approximately 20%.

Correspondingly CO_2_ emissions has been of great concern for a long time and there have been many relevant studies from the perspective of time, space and driving force models. From the perspective of time, although total carbon emissions continue to rise, the regional average carbon intensity has declined since 2005^4^. From the perspective of space, focusing on regional carbon emissions, spatial–temporal dynamics of carbon emissions and carbon sinks in Guangdong Province has been studied using the methodology based on land use/land cover data interpreted from continuous high-resolution satellite images and energy consumption statistics^5^. Results have shown that the carbon emissions in China are highly spatially unbalanced and that the distribution gap exhibits an increasing trend every year.

Regarding driving force model research, IPAT model was proposed in 1971^6^ to correlate the environmental pressure (I) with the three variables of the population (P), wealth (A), and environmental impact of unit energy consumption (T) to express the relationship between carbon emission indicators and macroscale variables. Subsequently, an improved STRIPAT model derived from the IPAT model has been applied to establish an exponential model with the population, economy, and technology as the main indicators, which enhanced the accuracy of the fitting and allowed the development of many variants of the model^7^. Structural decomposition analysis (SDA) and quantile regression have been applied to investigate changes in the carbon emission intensity in China^8^. A hybrid method combining variational mode decomposition (VMD), independent component analysis (ICA), and autoregressive integrated moving average (ARIMA) has been proposed to analyze the influencing factors of crude oil prices and to predict the future crude oil prices^9^. The fuzzy analytic hierarchy process has been used to analyze the e advantages and disadvantages of various renewable energy options in Turkey^10^, and results have indicated that wind energy is the best choice, followed by solar energy and biomass energy. Multiple linear regression^11^ has been used to fit and predict the development trend of the coal and coal chemical industries in China.

Of interest is whether China will achieve its emission reduction commitments. In order to be able to more accurately predict the future carbon emission intensity, we need to decompose the composition of carbon emissions, examine specific driving factors and try to deduce future reductions. However, the existing research has limitations, and driving factors have always been insufficiently considered to comprehensively reflect all influencing factors. Most of the driving factors of carbon emissions are universal macroscale factors (economic development, population situation, and technology). Furthermore, in the establishment of a driving force model, there must exist an optimal combination of qualitative and quantitative analysis. Purely qualitative analysis is greatly influenced by the subjective judgment of researchers, often leading to a lack of credibility, whereas purely quantitative analysis tends to limit scholars to specific data, while ignoring that carbon emissions is a comprehensive result closely related to national policies and regulations.

This study filled the above gap via the development of a comprehensive driving force model of the carbon emission intensity in China from 1990 to 2017. Firstly, a driving force model was established based on the three major sources of carbon emissions; furthermore, the carbon emission intensity and carbon emissions from 2018 to 2030 were predicted. Second, specific driving forces were chosen based on concrete indicators. Furthermore, the qualitative and quantitative factors were combined to establish a model for each source. In regard to the qualitive driving forces, such as policies, regulations, and laws, fitting functions were adopted used to quantify their effects.

## Methods

### Three main sources of carbon emissions

**Figure 2:**
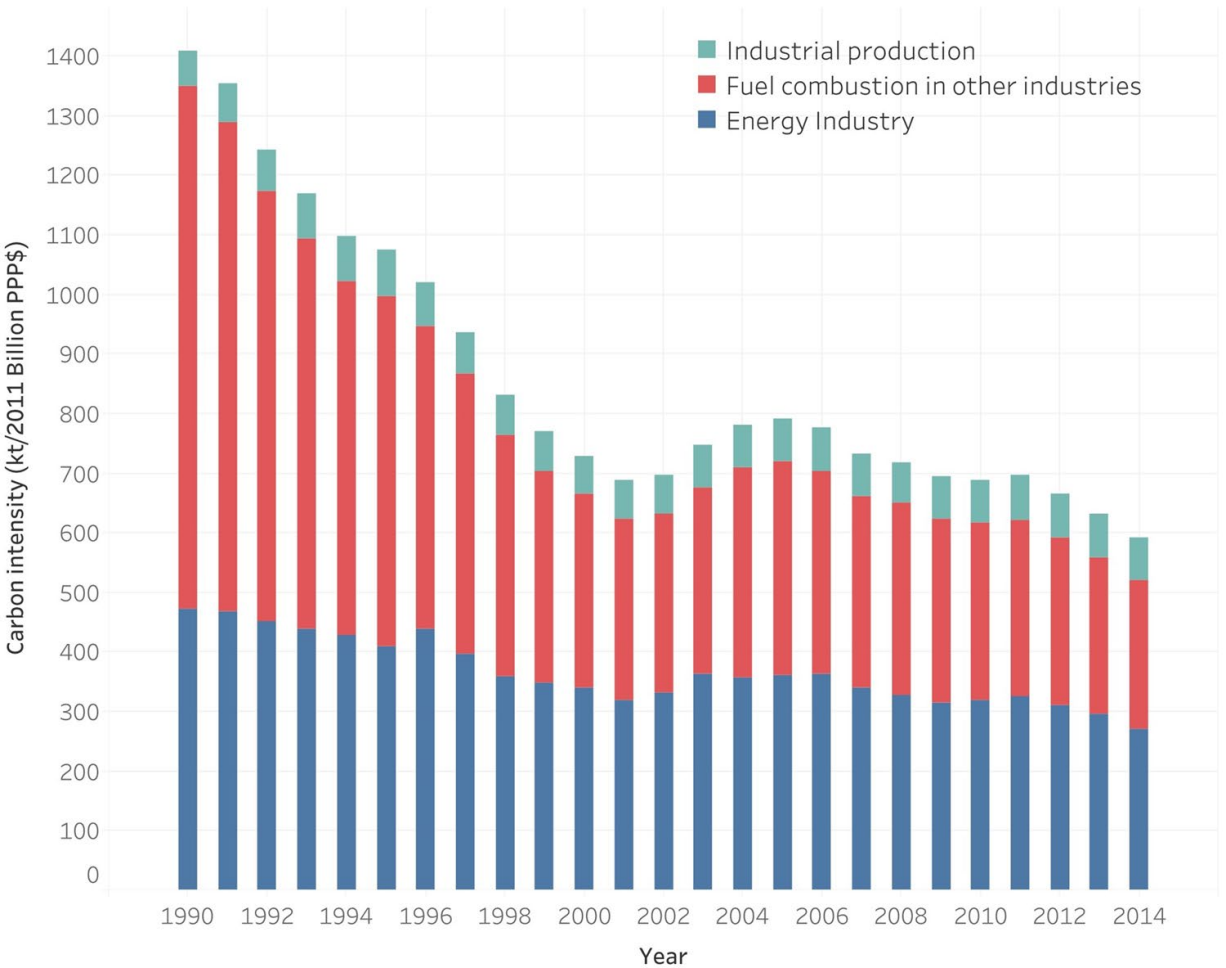
Carbon emission intensity composition in China.

According to World Bank data^3^, the total CO_2_ emission is 1.03 × 10^7^ kt while the total CO_2_ emissions in China originating from fuel combustion (the sum of CO_2_ emissions from solid fuel consumption, liquid fuel combustion and gaseous fuel combustion) are 9.04 × 10^6^ kt, which accounts for 87.92% of the total CO_2_ emissions. Among the CO_2_ emissions from fuel combustion, the CO_2_ emissions stemming from electricity and heat production occupy 52.25%. Except for fuel combustion, industrial production mainly the manufacture of cement, produces substantial CO_2_ emissions. Consequently, in this paper, three major emission sources are considered: the energy industry (the CO_2_ emissions stemming from electricity and heat production), fuel combustion in other industries, and industrial production (as shown in Fig. 2).

### Driving force model

This study assumes that the growth rate of the carbon emission intensity is a result of the superposition of the driving effects of various factors influencing the development of a given industry at a certain point in time^12–15^.

For each source of the carbon emissions, a multiple linear regression model is employed:

1 $$ Y_{t } = \mathop \sum \limits_{m = 1}^{i} \beta_{m} X_{m,t} + \varepsilon = \beta_{1} X_{1,t} + \beta_{2} X_{2,t} + \cdots + \beta_{i} X_{i,t} + \varepsilon , $$

where, $Y_{t }$ is the change rate of the carbon intensity at time t, $X_{m,t}$ is the change rate of the mth driving force at time t, $\beta_{m}$ is the regression coefficient of each driving force, i is the number of driving forces, and ε is the remainder.

Regarding each driving force, one or several representative indicator are chosen. In regard to quantitative indicators, their rate of change is directly calculated from their values. Regarding qualitative indicators such as policies, laws, and regulations, a fitting function is used to describe the change of their effectiveness over time. It simulates an exponential growth, similar to a logistic function. The basic function form is:

2 $$ y = k\frac{{1 - e^{ - rt} }}{{1 + e^{ - rt} }} t = 0,1,2 \ldots , $$

where k is the stable value of the function, r is the parameter describing the growth rate, and t is the execution time.

To correspond with quantitative data, the effectiveness of qualitative driving forces must also be converted into their annual change rate. The function is employed to define the rate of change at the corresponding time derivative.

3 $$ y^{\prime} = \frac{{2kre^{ - rt} }}{{\left( {1 + e^{ - rt} } \right)^{2} }} t = 0,1,2 \ldots $$

In order to compare the relative influence level among the various influencing factors, the regression coefficients were normalized.

4 $$ \beta ^{\prime}_{m} = \beta_{m} \times \frac{{\sigma_{{X_{m} }} }}{{\sigma_{Y} }}, $$

where $\beta ^{\prime}_{m}$ is the normalized regression coefficient of the of the mth driving force, $\beta_{m}$ is the regression coefficient of the driving force. $\sigma_{{X_{m} }}$ is the standard deviation of the driving force, and $\sigma_{Y}$ is the standard deviation of the dependent variable.

### Selection of the driving forces

Initially, for each source of the carbon emissions, several driving factors were selected (please refer to Table 1). By analyzing the significance of each driving factor (90% confidence interval), the effective driving forces finally obtained are as follows (the one-tailed T test was used).

**Table 1:** Selection of the driving forces.

Emission source	Driving force	Indicator	Significance	P value
Energy industry	Renewable energy development	Water, wind and nuclear power consumption ratio	×	
	Market demand changes	Total energy consumption	√	$1.7021 \times 10^{ - 5}$
	Energy industry regulations	Relevant policies	√	0.0327
	Industrial structure reforms	Relevant policies	√	0.0537
	Industrial technology innovation	Energy processing conversion total efficiency	√	0.0012
	Accidental events	Accidental events	×	
Fuel combustion in other industries	Energy consumption intensity	Energy intensity	√	$2.7999 \times 10^{ - 8}$
	New-energy vehicle development	Number of new energy vehicles	×	
	Energy conservation and emissions reduction policies	Relevant policies	√	0.00565
	Energy conservation technology promotions	Relevant policies	×	
	Industrial transformation and upgrading	Relevant policies	×	
	Accidental events	Accidental events	×	
Industrial production	Metal production	Crude steel annual output	×	
	Nonmetallic mineral production	Cement production annual output	√	$9.3181 \times 10^{ - 9}$
	Chemical industry production	Ammonia annual output	×	
	Investment in scientific research	Research and experimental development expenditures	×	
	Promotion of technological innovation	Patent grants	×	
	Emission reduction policies	Relevant policies	×	
	Accidental events	Accidental events	×	

Six key driving forces of the energy industry were selected, namely renewable energy development, market demand changes, energy industry regulations, industrial structure reforms, industrial technology innovation, and accidental events. In energy industry, renewable energy has grown fast and the ever-increasing proportion of renewable energy has also reduced the carbon emission intensity of the energy industry. According to the clean energy installed capacity and carbon emission intensity of the five largest power generation companies in China, the decline in carbon emission intensity and the increase in clean energy installed capacity are positively correlated (please refer to Table 2). The market demand change, i.e., the total energy consumption obviously exerts a significant impact on total carbon emissions.

**Table 2:** Comparison of the clean energy installed capacity and power generation carbon intensity of five power generation companies^16^.

Power generation company	Clean energy installed capacity ratio (%, 2015)	Power generation carbon intensity
China Huaneng	28.8	670.6
China Datang Corporation	31.48	653.4
China Huadian Corporation	37.1	591.3
China Guodian Corporation	29.9	670.2
State Power Investment Corporation	40	568.3

The energy industry has been completely operated by the government in the past and relevant regulatory laws have been implemented relatively late. After the establishment of the National Energy Administration in 2008, relevant laws and regulations have been introduced. Constant market-oriented reforms have also created problems of excessive development and rapid production capacity growth. Therefore, it is essential to promote industrial structure reform. In regard to these two driving forces, a series of relevant policies have been selected as specific indicators. In the energy industry, technological innovation mainly occurs in two fields: energy processing and energy transportation. The selected indicator, technological innovation improves the energy conversion efficiency and decreases the energy production costs and pollution emissions in the field of energy processing and can reduce energy consumption and regulates the energy distribution in the field of energy transportation reserves. Finally, accidental events were selected for all three main sources of carbon emissions.

In terms of fuel combustion in other industries, the energy consumption intensity, new-energy vehicle development, energy conservation and emissions reduction policies, energy conservation technology promotion, industrial transformation and upgrading, and accidental events were identified as driving forces. The energy intensity is a comprehensive indicator as well as one of the most closely related and most influential indicators of the carbon emissions of fuel combustion. The transportation industry is the main force for the consumption of liquid fuels. After the launch of the Eleventh Five-Year Plan, preferential policies for the promotion of new-energy vehicles were constantly introduced and the relative investments in core scientific research increased. The annual output change of new energy vehicles (including hybrid vehicles) was selected to portray this driving force. As the ecological crisis continues to intensify, China has paid increasing attention to the use of energy and the discharge of pollution. China has also begun to tighten the emissions standards for companies and regions and has advocated cleaner production. With the introduction of energy-saving and emissions reduction techniques, companies with high pollution and high energy consumption are finding it increasingly difficult to survive. Therefore, the invention and use of innovative techniques has attracted increasing interest. To achieve low-carbon development, all industries must be transformed and upgraded. In regard to these three qualitative driving forces, relevant policies were selected as specific indicators.

Regarding industrial production, seven driving forces were chosen: production of metal products, production of nonmetallic mineral products, production of the chemical industry, investment in scientific research, promotion of technological innovation, emissions reduction policies, and accidental events. The production of metal products is a typical high-energy and high-emission process. The most important source of the carbon emissions originating from the production of metal products is steel production. China is not only a major steel producer and consumer, but also a major exporter. In 2018, the crude steel output of China reached 928 million tons. In 2017, the export volume of steel products was 75.41 million tons, while the import volume was only 13.3 million tons. The process of nonmetallic mineral production was the largest source of the carbon emissions of industrial production, including cement, flat glass, and lime. Of these, the cement industry occupied the vast majority. Hence, the annual rate of change in the cement production industry provided by the National Bureau of Statistics database was chosen as an indicator. Chemical industries include the coal chemical industry, and petrochemical industry, among others. Compared to metal or nonmetal production, their carbon emission scale is slightly lower with a more diverse and complicated emission mechanism. Among the various subsectors of the chemical industry, coal-based ammonia is a typical representative. The annual rate of change in synthetic ammonia was selected as an indicator. As the carbon emissions of industrial production processes largely depend on various technological processes, scientific research is also an important driving force. Therefore, the annual rate of change in research and experimental development expenditures was chosen as an indicator of the scientific research input. As mentioned above, scientific research and technological innovation exert a significant impact on the carbon emissions of industrial production processes. Research and experimental development expenditures constitute part of the investment. Furthermore, from the perspective of the technological output, patent grants are another important reference data. To further improve the driving system, process emissions reduction techniques and policies were also considered.

The influence range of carbon emission indicators is extensive and the sources are complicated; hence, response to accidental events is also complex. In the case of short-term dramatic changes, considering the impact of accidental events plays a more complementary role among the carbon intensity indicators. In this paper, three dimensions of accidental events were selected: the hosting of major international events (2008 Beijing Olympic Games, the 2011 Shanghai World Expo, etc.), the period of major economic fluctuations within the international scope (the Asian financial crisis from the end of the last century to the beginning of this century and the beginning of the global financial crisis in 2008), and major natural disasters or outbreaks (the SARS epidemic in China in 2003 and the Sichuan earthquake in 2008 along with its resultant disasters).

### Data

Emission data were derived from the world development indicators in the World Bank database, which provided the total carbon emissions in China until 2014^17^; By comparing the World Bank data and data provided by the Global Carbon Project^18^, the CO_2_ emissions data for the same source each year differ by an approximate percentage, therefore the carbon emission data from 2015 to 2017 may be supplemented. GDP data were derived from the 2011 US dollar-based purchasing power parity GDP of China provided in the World Bank database^19^. Regarding the data of each specific driving force, national statistical yearbooks from 2000 to 2018 were referenced^20^.

In regard to qualitative indicators, the parameter k is the stable value and is decided by the long-term effect of a specific policy. In this paper, if the policy is a law, we set k = 1; if it is an administrative measure, we set k = 0.5. The parameter r represents the rate of change of a policy and is determined according to the actual length of time a specific policy takes effect. Generally speaking, the more influential a policy is, the longer it will take to be fully effective, and this leads to a smaller value of r. The specific values of k and r are shown below (please refer to Table 3).

**Table 3:** Value of k and r for each policy.

Driving force	Indicator	k	r
Energy industry regulations	Establishment of the National Energy Administration	0.5	0.2
	Regulations such as Energy-saving Power Generation Measures	0.5	0.5
	Energy Conservation Law of the People's Republic of China	1	0.2
	Guiding Opinions on Promoting the Scientific Development of the Coal Industry	0.5	0.5
	Notice of the National Energy Administration on promoting the orderly development of coal power in China	0.5	0.5
	Measures by the Administration of Credit Evaluation of Energy Market Subjects (Trial)	0.5	1
	12,398 Energy Regulatory Hotline Complaint Reporting Guide	0.5	1
Industrial structure reforms	Power System Reform Program	0.5	0.5
	Coal price linkage	0.5	0.5
	Renewable energy law	1	0.2
	Forced shutdown of small thermal power units and operations	0.5	0.5
	Energy Development Strategy Action Plan (2014–2020)	0.5	0.5
	Energy Technology Revolution and Innovation Action Plan (2016–2030)	0.5	0.5
	Notice on the Pilot Project of Distributed Generation Marketization	0.5	0.5
Energy conservation and emission reduction policies	China Energy Conservation Technology Policy Outline		
	Energy Conservation Law of the People's Republic of China	1	0.2
	Law of the People's Republic of China on Cleaner Production Promotion	1	0.2
	Comprehensive Energy-saving and Emission Reduction Work Plan	0.5	0.5
	Work Arrangement for Energy-saving and Emission Reduction	0.5	0.5
	Law of the People's Republic of China on the Promotion of the Circular Economy	1	0.2
	Application for new-energy demonstration cities and industrial parks	0.5	0.5

## Results

### Energy industry carbon emission driving force model

The CO_2_ emissions of the energy industry indicate the CO_2_ generated during electricity and heat production. Four key driving forces of the energy industry were selected, namely, the market demand change, energy industry regulation, industrial structure reforms and industrial technology innovation (please refer to Table 4). Obviously, the market demand is directly proportional to the total output of the energy industry, while industrial technology innovation represents the efficiency of the energy production process. Due to the characteristics of a high energy consumption and high pollution, the energy industry requires effective regulations and continuous industrial structure reforms.

**Table 4:** CO_2_ emissions driving forces in energy industry. (Data source: calculated fitting coefficients).

Driving force	Indicator	Time	Normalized coefficients
Market demand change	Total energy consumption change rate	Full cycle	1.1181
Energy industry regulations	Establishment of the National Energy Administration	2008	0.4651
	Regulations such as Energy-saving Power Generation Measures	2007	
	Energy Conservation Law of the People's Republic of China	2007	
	Guiding Opinions on Promoting the Scientific Development of the Coal Industry	2015	
	Notice of the National Energy Administration on promoting the orderly development of coal power in China	2016	
	Measures by the Administration of Credit Evaluation of Energy Market Subjects (Trial)	2017	
	12,398 Energy Regulatory Hotline Complaint Reporting Guide	2017	
Industrial structure reforms	Power System Reform Program	2002	− 0.4987
	Coal price linkage	2004	
	Renewable energy law	2005	
	Forced shutdown of small thermal power units and operations	2007	
	Energy Development Strategy Action Plan (2014–2020)	2014	
	Energy Technology Revolution and Innovation Action Plan (2016–2030)	2016	
	Notice on the Pilot Project of Distributed Generation Marketization	2017	
Industrial technology innovations	Energy processing conversion total efficiency change rate	Full cycle	− 0.3508

The market demand change and industrial structure reforms with positive coefficients indicate that they impose a positive effect on the carbon emission intensity while the other two driving forces with negative coefficients yield the opposite impact. Obviously, the higher the market demand for energy is, the higher the carbon intensity. Conversely, industrial technology innovations improve the energy conversion efficiency and industrial structure reforms allow more clean energy to be used. However, strengthened energy regulations also lead to an increased carbon emission intensity. This may occur because the current energy regulatory system in China still has flaws, such as the absence of a basic energy law and conflicts between laws and regulations^21^. According to the absolute value of the normalized coefficients, the contributions of each driving force follow the order of the market demand change, industrial structure reforms, energy industry regulations and followed by industrial technology innovations.

### Fuel combustion in other industries

The CO_2_ emissions originating from fuel combustion in other industries indicate the emissions of fuel combustion except for those of heat and electricity production. Initially, several driving forces were considered, such as the energy consumption intensity, new-energy vehicle development, and industrial transformation and upgrading (please refer to Table 5). However, only the energy consumption intensity and energy conservation and emission reduction policies were significant (90% confidence interval).

**Table 5:** CO_2_ emissions driving forces in fuel combustion in other industries. (Data source: calculated fitting coefficients).

Driving force	Indicator	Time	Normalized coefficients
Energy intensity	Energy intensity change rate	Full cycle	0.8030
Energy conservation and emission reduction policies	China Energy Conservation Technology Policy Outline	1990–2017	0.3243
	Energy Conservation Law of the People's Republic of China	1997–2017	
	Law of the People's Republic of China on Cleaner Production Promotion	2003–2017	
	Comprehensive Energy-saving and Emission Reduction Work Plan	2007–2017	
	"Work Arrangement for Energy-saving and Emission Reduction	2008–2017	
	Law of the People's Republic of China on the Promotion of the Circular Economy	2008–2017	
	Application for new-energy demonstration cities and industrial parks	2012–2017	

The increase in energy intensity and the implementation of energy-saving and emission reduction technologies both increase the intensity of carbon emissions, and the impact of energy intensity is great. Regarding the reason why policies related to energy conservation and emission reduction will cause an increase in carbon emission intensity, a hypothesis is proposed: these policies are not driving forces of CO_2_ emissions but are rather driven by them. Fuel combustion emits a large amount of carbon dioxide, as well as other exhaust gases and wastewater. The large amount of waste emissions resulted various energy conservation and emission reduction policies. The correlation coefficient of the sulfur dioxide emissions in China and the energy conservation and emissions reduction policies is 0.7050, and the correlation coefficient of smoke and dust emissions is 0.7187 (data retrieved from national statistical yearbooks^20^). These correlation coefficients reflect the close relationship between them.

### Industrial production carbon emission driving force model

The carbon emissions originating from industrial processes are total carbon emissions minus those originating from fuel combustion. Metal production, nonmetallic mineral production, chemical industry production, investment in scientific research, promotion of technological innovation, emissions reduction policies and accidental events were analyzed but only non-metallic mineral production achieved significance (please refer to Table 6).

**Table 6:** CO_2_ emissions driving forces in industrial production.

Driving force	Indicator	Time	Normalized coefficients
Nonmetallic mineral production	Annual rate of change in cement production	Full cycle	1

These results indicate that the impact of the cement industry far exceeds that of the other metal and chemical industries, and the impact of related policies is limited.

### Performance

Since the 2015–2017 carbon emissions data of industrial production cannot be relatively accurately determined, the modeling period of industrial production is selected as 1991–2014. Due to the general quality of the driving force data in the energy industry model from 1995 to 1998, the modeling period of energy industry is selected as 1999–2017. According to cross-validation, the performance of these modeling intervals is the best.

To evaluate the modeling performance, the significance F and adjusted R-squared values are considered (please refer to Table 7). The R-Square is defined below, which represents the degree to which a given independent variable explains the dependent variable.

5 $$ R^{2} = 1 - \frac{{\sum \left( {Y_{actual} - Y_{predict} } \right)^{2} }}{{\sum \left( {Y_{actual} - Y_{mean} } \right)^{2} }}. $$

**Table 7:** Performance.

	Significance F	R^2^	Adjusted R-squared Value	LOOCV Adjusted R_cv_-squared value	Period
Energy industry	7.2974E−05	0.8043	0.7483	0.4677	1999–2017
Fuel combustion in other industries	3.2884E−08	0.7621	0.7423	0.5823	1991–2017
Industrial production	9.3181E−09	0.7833	0.7734	0.7313	1991–2014

To eliminate the impact of the increase in the number of independent variables, the adjusted R-Squared value is considered. This expresses the marginal effect of each newly added independent variable on model fitting.

6 $$ {\text{Adjusted R{-}Squared value}} = 1 - \frac{{\left( {1 - R^{2} } \right)\left( {n - 1} \right)}}{n - p - 1}, $$

where n is the number of sample and p is the number of independent variables.

To avoid overfitting, leave-one-out cross-validation is applied. In a data set of n samples, n − 1 samples are adopted as the training set and the remaining sample is adopted as the test set. The concept of the adjusted R-squared value is also applied in leave-one-out cross-validation.

7 $$ R_{cv}^{2} = 1 - \frac{{\sum \left( {Y_{test} - Y_{predict\_training} } \right)^{2} }}{{\sum \left( {Y_{actual} - Y_{mean} } \right)^{2} }}, $$

8 $$ {\text{Adjusted}}\,{ }R_{cv} { }\,{\text{squared }}\,{\text{value}} = 1 - \frac{{\left( {1 - R_{cv}^{2} } \right)\left( {n - 1} \right)}}{n - p - 1}. $$

The result of cross-validation demonstrates that in the model of energy industry and fuel combustion in other industries there is a certain degree of overfitting but it is acceptable, while in the industrial production model there is almost no overfitting.

**Figure 3:**
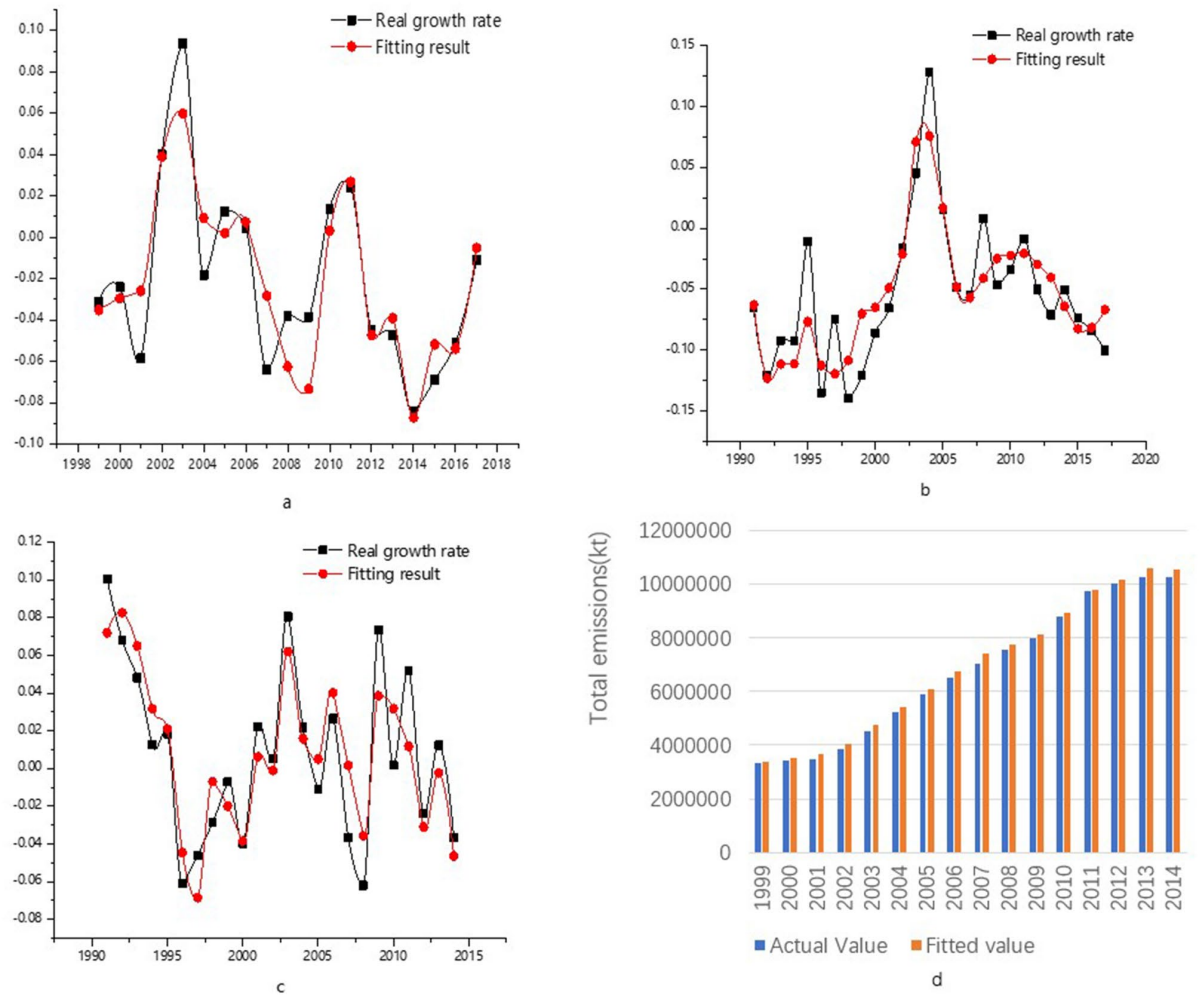
Regression analysis results [(**a**) energy industry, (**b**) fuel combustion in other industries, (**c**) industrial production, (**d**) total emissions]. (Data source: performance of modelling).

Figure 3 shows the fitting result of the growth rate of each sector and total emissions. The change trend of the change rate is basically fitted. Regarding the total emissions from 1999 to 2014, the average relative error is approximately 3%.

### Forecast of the carbon intensity and total CO_2_ emissions from 2018 to 2030

Based on the established model, forecasts of the Chinese carbon intensity and emissions from 2018 to 2030 were obtained. This paper first predicted the value of each driving force and then calculated the corresponding carbon intensity and total carbon emissions for each year. Regarding quantitative data, the predicted data were mainly obtained via regression analysis with adjustments according to future expectation. Regarding qualitative data (policies, regulations, laws, etc.), the same fitting function was applied and the future impacts of current policies were calculated.

#### Forecast of energy consumption and energy intensity

Except for a slight increase in energy intensity from 2003 to 2005, the energy intensity showed a continuous downward trend. Compared to 1990, the energy intensity in 2018 decreased by 63.96%. The sustainability of this trend is a key factor in the future trends of the carbon emissions intensity and total emission change. An adaptive genetic algorithm energy demand estimation (AGAEDE) optimal model has been proposed to improve the efficiency of energy demand prediction and the simulation results show that the energy demand in China will reach 5.23 billion standard tons of standard coal equivalent (TCE) in 2020^22^. The ADL-MIDAS model has been applied to predict the energy demand in China and its structure and the result shows that by 2020, the total energy demand in China reached approximately 4.65 billion TCE^23^. An improved energy demand forecasting model was built based on the autoregressive distributed lag (ARDL) bounds testing approach and adaptive genetic algorithm (AGA) considering GDP, economic structure, urbanization, and technological progress and the results demonstrate that China will demand approximately 4.9, 5.6, and 6.1 billion standard tons of coal equivalent in 2020, 2025, and 2030, respectively^24^.

Standard and Poor's stated in a report the Great Game and an Inescapable Slowdown that after 40 years of rapid economic growth, the average annual economic growth rate in China is expected to decline to 4.6% over the next ten years due to demographic changes, deleveraging, economic rebalancing from manufacturing to services, etc.^25^. Table 8 shows the assumptions of future energy consumption and GDP growth.

**Table 8:** Assumption of energy consumption and GDP prediction.

		2018	2020	2022	2024	2026	2028	2030
Energy consumption (billon TCE)		4.64	4.96	5.19	5.44	5.67	5.91	6.18
GDP growth rate (%)		6.7	6.0	5.7	5.3	4.9	4.6	4.1

#### Forecast of the cement output

The output of cement directly affects the carbon dioxide emissions of the industrial production process. It has declined with fluctuations after reaching its peak in 2014, and showed a rising trend in 2019. According to USGS.gov^26^, in 2016, the cement consumption per capita was 1.64 tons, 6.85 times of that of the United States and 3.82 times of that of Japan. An extended STIRPAT model was exploited to examine the effect of the population, gross domestic product (GDP) per capita, cement consumption intensity, fixed investments and urbanization level of China from 2005 to 2013 and the results demonstrate that fixed investments and the GDP per capita are the decisive driving factors of cement consumption^27^. The period of rapid growth of the fixed investments in China has passed and by 2019 the fixed investment had decreased by 13.13% cover the 2018 level. The China Cement Association has released the Cement Industry Capacity Removal Action Plan (2018–2020), indicating that overcapacity of cement has seriously hindered the sustainable development of the industry, and decreasing the capacity will be the main battle of the industry during the 13th Five-Year Plan. The goal is to reduce the clinker production capacity by 392.7 million tons and close 540 cement grinding station companies by 2020. The national concentration of the clinker production capacity of the top 10 largest enterprise groups is higher than 70%, and the concentration of the cement production capacity reaches 60%. In summary, the future output of cement will show a gradual decline (please refer to Table 9).

**Table 9:** Assumptions of the cement output.

		2018	2020	2022	2024	2026	2028	2030
Cement output(kt)	2,207,707		2,326,500	2,280,203	2,234,827	2,190,354	2,146,766	2,104,045

#### Results

**Figure 4:**
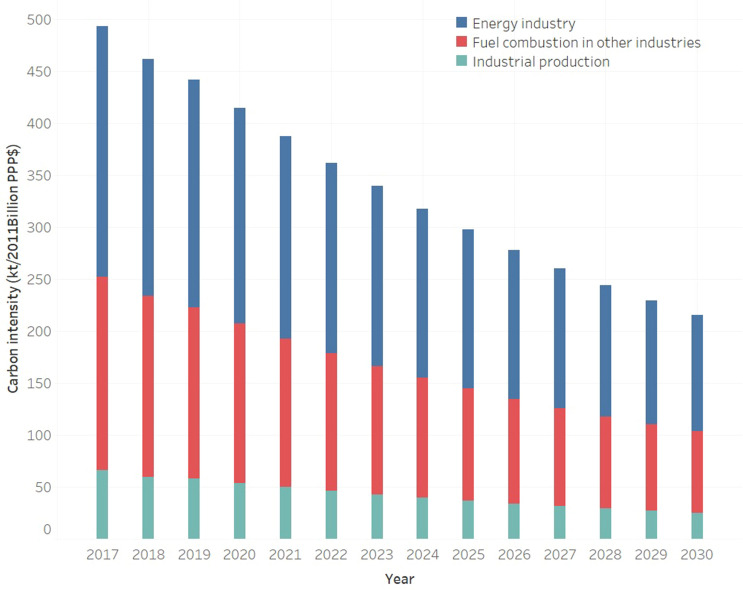
CO_2_ emission intensity forecast from 2018 to 2030. (Data Source: prediction result).

The carbon intensity for all three carbon emission sources showed a downward trend in during the period from 2018 to 2030 (please refer to Fig. 4). The energy industry, fuel combustion in other industries, industrial production and total carbon intensity achieved reductions of 51.06%, 54.81%, 57.97% and 53.37% respectively.

Compared to 2017, the proportion of the energy industry will slightly rise to 51.79% from 48.94%, while the proportion of the other two sources will slightly decrease.

**Figure 5:**
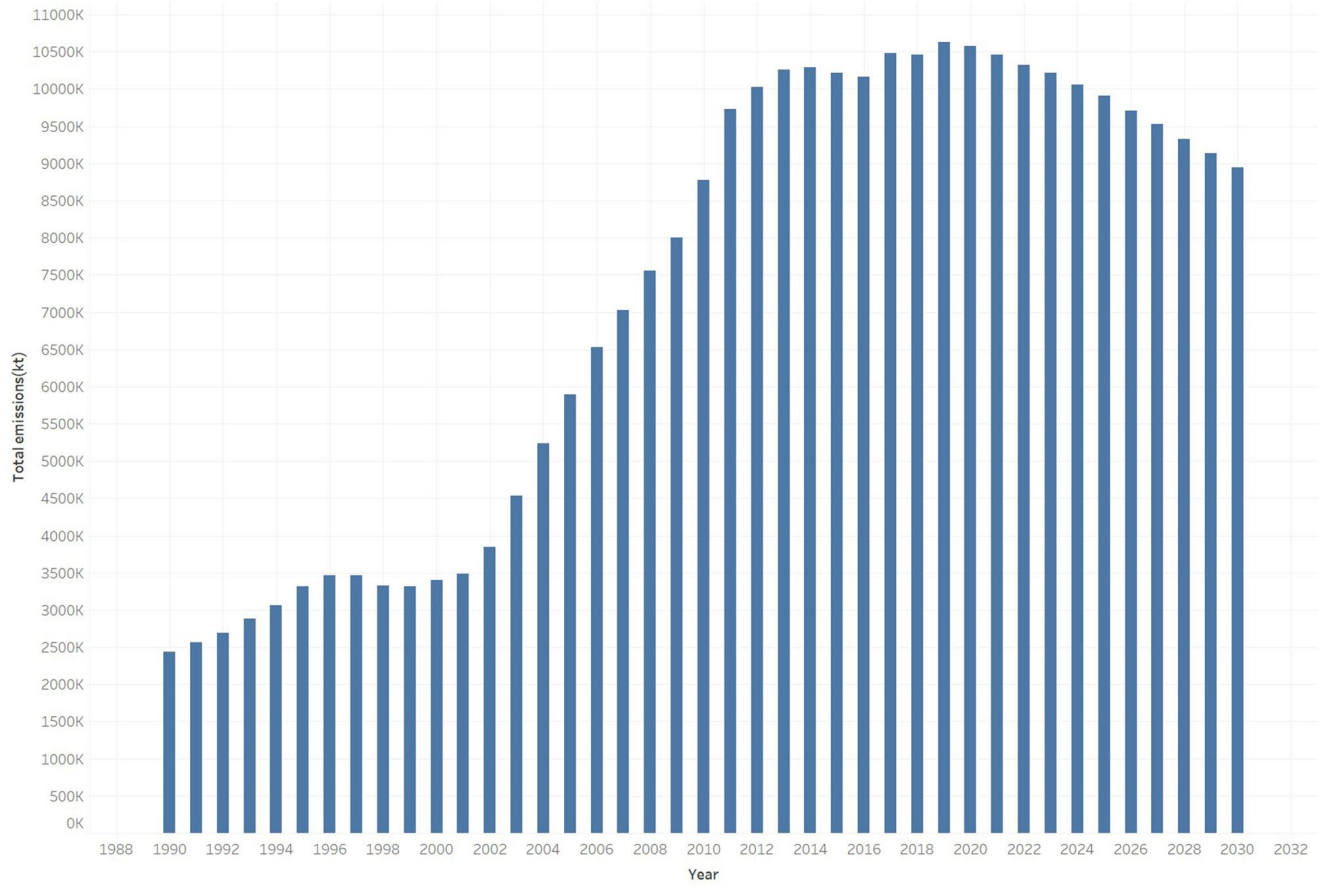
Total CO_2_ emissions (forecast from 2018 to 2030). (Data source: prediction result).

Considering the total CO_2_ emissions, the total CO_2_ emissions in China have gradually increased after 1990, fluctuated in 1998 and then rapidly increased to a peak in 2014. After a slight decline from 2015 to 2016, it rose in 2017 and fluctuated and will peaked again in 2019. After 2019 there was a downward trend and by 2030 it will return to 8,947,488 kt, almost similar to that in 2010 (please refer to Fig. 5). Whether such a decline may be achieved depends to a large extent on whether the energy intensity and cement output are reduced.

In the energy industry, industrial structure reforms contribute 73.5% of carbon intensity reduction while industrial technology innovations contribute the rest. Overall, the energy industry, fuel combustion in other industries and industrial processes will reduce carbon intensity by 130.10, 107.06 and 41.25 kt/2011 Billon PPP$, respectively. From the perspective of the specific driving force, the contributions of the driving forces to the estimated carbon emission reduction in descending order are the energy intensity, industrial structure reform, industrial technology innovation and nonmetallic mineral production.

Under current policies, the carbon emissions intensity in China will continue to decline, and the total carbon emissions will slowly decline. To further reduce CO_2_ emissions, there are three main points. First, the government should focus on adjusting the energy structure to reduce the energy intensity. As the rise in the use of coal exhibits a strong correlation with CO_2_ emissions, coal-to-oil and coal-to-natural gas should be the direction of future efforts. Second, the CO_2_ production per unit of cement should be reduced. This may be improved by reducing the energy consumption or utilization of clean energy and reducing the proportion of clinker proportion. Third, more policies must be introduced.

## Discussion

### Sources of errors

The adjusted R-squared value of the model is not high, indicating that there is room for further improvement in terms of the model fitting degree and predictive potential. This may be due to the complexity of the problem, and determination of the most influential factor is a direction of future effort. Nevertheless, significance analysis of each driving factor and the performance of the fitting results indicate that the results provide a certain guiding significance for the reduction in the carbon intensity and total CO_2_ emissions. The forecast error mainly stems from the lack of future policies and the uncertainty of future economic development. Since the current model only includes the impact of current policies, future carbon emissions may be overestimated. In addition to this, the carbon emission intensity is predicted first, then the total carbon emissions are obtained by predicting GDP, leading to the result being sensitive to future GDP.

### Turning point of 2014

**Figure 6:**
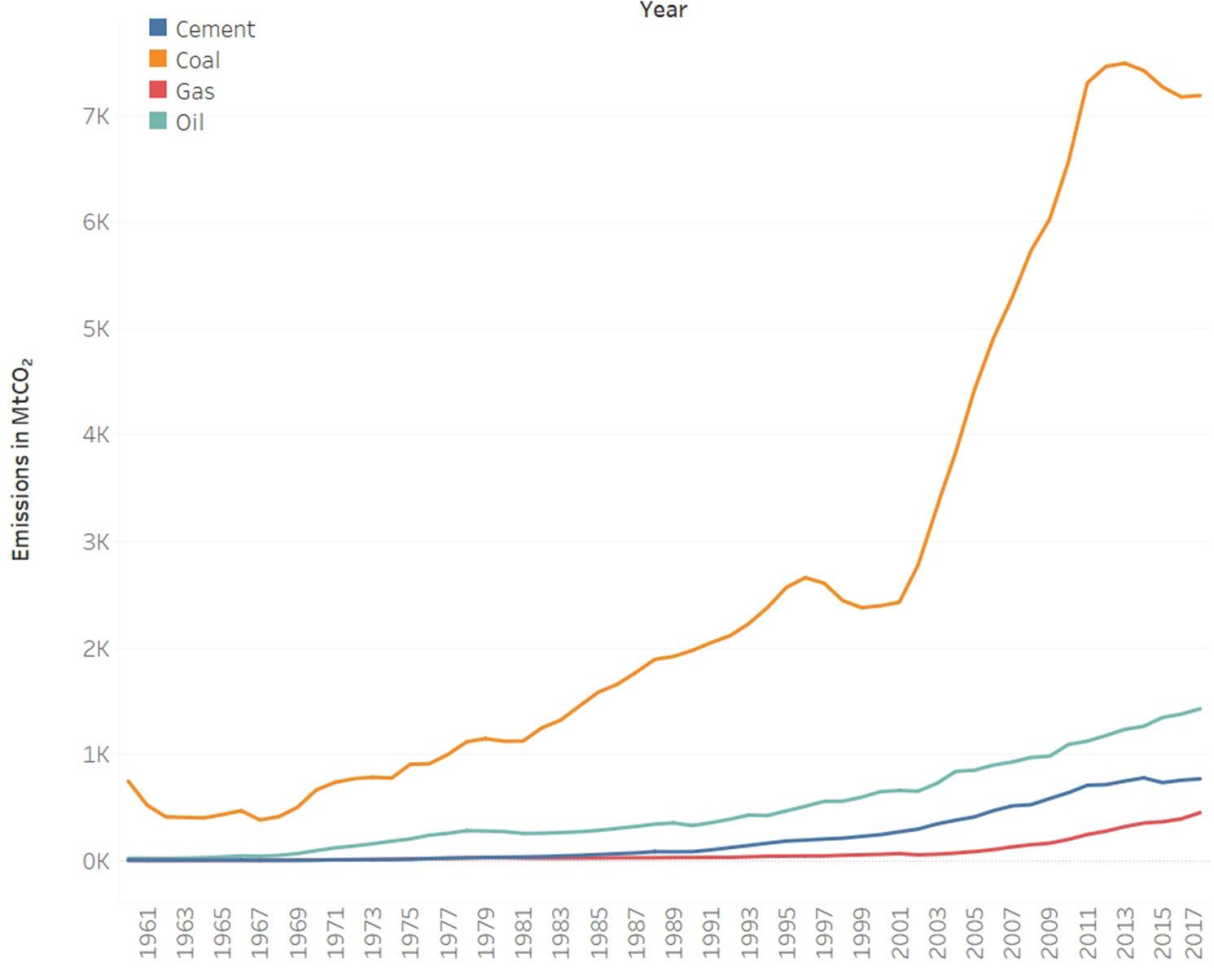
Annual fossil CO_2_ emissions in China.

In regard to the reasons for 2014 is a turning point of the total emissions, the energy intensity played a major role. With increasing total energy consumption, the decrease in energy intensity mainly stemmed from the improvement in the energy structure. According to the Global Carbon Project^18^, the CO_2_ emissions originating from coal increased between 1960 and 2013 and declined until 2016. However, the emissions stemming from oil, gas, and cement gradually increased. The reduction in coal use and improvement in the type of coal used (less the unit carbon emissions) played an important role in the decline in carbon intensity since 2015, while the simultaneous increase in 2017 led to a rebound in the carbon intensity in 2017, which is consistent with our findings (as shown in Fig. 6). The trends of the total carbon emissions are consistent with existing data.

### Comparison to the Paris agreement 1.5 °C scenario

According to the Emissions Gap Report 2019^28^, scientists agree that to limit the global temperature rise to 1.5 °C, emissions must rapidly decline to 25 gigatons by 2030. Collectively, if commitments, policies and actions deliver a 7.6% emissions reduction every year between 2020 and 2030 worldwide, we may limit global warming to 1.5 °C. According to our research, from 2020 to 2030 the average reduction in emissions in China is approximately 1.66% which indicates that more efforts should be made. However, achieving a CO_2_ emissions peak before 2030 is optimistically predicted.

To further reduce the carbon emissions of China, polices should focus more on improving energy structure and eliminating any backward production capacity. The improvement in the energy structure not only includes more clean energy in energy industry, but also includes a decreased fossil energy use at the energy use stage. In the energy industry, the increase of renewable energy should be strongly encouraged with relevant subsidies. In regard to fuel combustion in other industries, the use of new energy vehicles and introduction of energy-saving building materials may reduce the use of fossil energy. Although the capacity and output of cement are gradually decreasing, we still must pay attention to the carbon emissions caused by unit cement production. Overall, the formulation of policies should be carried out under a unified plan, and they should not conflict with each other.

### Impacts of COVID-2019

Since the outbreak of COVID-19 has greatly affected China and the world in 2020, and was not considered in original forecast model, it is necessary to analyze its short-term influence. The outbreak affected the industrial production and energy consumption of China in the first quarter of 2020. According to the National Bureau of Statistics, in the first quarter of 2020 the annual growth of the total energy consumption fell 2.8%^29^ and the annual growth of the GDP fell 6.8%^30^. The cement output in the first quarter was 299 million tons, a decrease of 23.93%, the largest decline since the start of this century^31^. Although there later changes may occur, the carbon emissions in 2020 should be lower than expected. It is estimated that the total carbon emissions in 2020 will reach 9,058,662.813 kt, which are 14.3% lower than in 2019. If the epidemic persists for a longer period of time, the total carbon emissions will further decrease, which will help achieve the Paris Agreement goal. In the long run, the impact of the epidemic may accelerate the change of lifestyles. Working from home, and online meetings and education may help people actively examine the possibility of a lower-carbon lifestyle actively. If people find that online collaboration is less expensive and more efficient than meeting in the field during this period, then this may become a powerful driver for carbon emissions reduction.

## Conclusion

This paper conducted a study of the carbon emission intensity in China from 1990 to 2017. A comprehensive driving force model was established concerning both qualitative and quantitative factors and a prediction of the total carbon emissions in China from 2018 to 2030 was obtained.

There are three main sources of the carbon emissions in China: energy industry, fuel combustion in other industries, and industrial processes. Specific driving forces and corresponding indicators, instead of macroscale factors such as technology and population were selected to establish the model. In the energy industry, the market demand change, energy industry regulations, industrial structure reforms and industrial technology innovations yielded significance. In fuel combustion in other industries, the energy intensity and energy conservation and emission reduction policies played a major role. In industrial production, the impact of nonmetallic mineral production (cement production) far exceeded those of the other influencing factors. Among the quantitative factors, the market demand change, energy intensity and nonmetallic mineral production exerted a positive influence on CO_2_ emissions and industrial technology innovations produced the opposite impact. Concerning the qualitative factors, industrial structure reforms helped reduce CO_2_ emissions but energy industry regulation led to a further increase in CO_2_ emissions. According to the fitted numerical results, energy conservation and emissions reduction policies yielded the opposite effect to what was expected. Through further analysis, it was found that the value of this driving force exhibits a notable correlation with the national emissions of several major pollutants. Therefore, this driving force was more likely to be the result of CO_2_ emissions rather than their cause.

From 2018to 2030, the carbon intensity and total CO_2_ emissions will experience a downward trend. By 2030, the total CO_2_ emissions will return to the 2010 level. Moreover, 2015 has become a turning point in CO_2_ emissions, mainly due to the improvement in the energy structure, and the decline in the proportion of coal use is an important indicator. From 2018 to 2030, the most important driving force of emission reduction is energy intensity. followed by industrial structure reforms, industrial technology innovation and nonmetallic mineral production.

According to the current emissions reduction efficiency, the goals of the Paris Agreement may not be achieved, but the development of renewable energy, the improvement of technology for emissions reduction, and the potential change in lifestyle are encouraging. Current policies to reduce carbon emissions should focus on improving the energy structure and thereby reducing the energy intensity. The formulation of policies should be carried out under a unified plan and policies should not conflict with each other. Although the capacity and output of cement are gradually decreasing, we must pay attention to the carbon emissions per unit cement production.
